# Proinflammatory role of amphiregulin, an epidermal growth factor family member whose expression is augmented in rheumatoid arthritis patients

**DOI:** 10.1186/1476-9255-5-5

**Published:** 2008-04-27

**Authors:** Shoji Yamane, Satoru Ishida, Yukie Hanamoto, Ken-ichi Kumagai, Riako Masuda, Konagi Tanaka, Noriyuki Shiobara, Noriko Yamane, Toshihito Mori, Takuo Juji, Naoshi Fukui, Tsunetoshi Itoh, Takahiro Ochi, Ryuji Suzuki

**Affiliations:** 1 Clinical Research Center for Allergy and Rheumatology, National Hospital Organization, Sagamihara National Hospital, Sakuradai 18-1, Sagamihara, Kanagawa 228-8522, Japan; 2 Discovery Research Laboratories, Shionogi & Co., Ltd., 3-1-1, Futaba-cho, Toyonaka, Osaka 561-0825, Japan; 3 Department of Immunology and Embryology, Tohoku University School of Medicine, 2-1 Seiryo-Machi, Aoba-ku, Sendai 980-8575, Japan

## Abstract

**Background:**

The epidermal growth factor (EGF) and EGF receptor (EGFR) families play important roles in the hyperplastic growth of several tissues as well as tumor growth. Since synovial hyperplasia in rheumatoid arthritis (RA) resembles a tumor, involvement of the EGF/EGFR families in RA pathology has been implied. Although several reports have suggested that ErbB2 is the most important member of the EGFR family for the synovitis in RA, it remains unclear which members of the EGF family are involved. To clarify the EGF-like growth factors involved in the pathology of RA, we investigated the expression levels of seven major EGF-like growth factors in RA patients compared with those in osteoarthritis (OA) patients and healthy control subjects.

**Methods:**

The expression levels of seven EGF-like growth factors and four EGFR-like receptors were measured in mononuclear cells isolated from bone marrow and venous blood, as well as in synovial tissues, using quantitative RT-PCR. Further evidence of gene expression was obtained by ELISAs. The proinflammatory roles were assessed by the growth-promoting and cytokine-inducing effects of the corresponding recombinant proteins on cultured fibroblast-like synoviocytes (FLS).

**Results:**

Among the seven EGF-like ligands examined, only amphiregulin (AREG) was expressed at higher levels in all three RA tissues tested compared with the levels in OA tissues. The AREG protein concentration in RA synovial fluid was also higher than that in OA synovial fluid. Furthermore, recombinant human AREG stimulated FLS to proliferate and produce several proinflammatory cytokines, including angiogenic cytokines such as interleukin-8 and vascular endothelial growth factor (VEGF), in a dose-dependent manner. The VEGF mRNA levels in RA synovia and VEGF protein concentrations in RA synovial fluid were significantly higher than those in the corresponding OA samples and highly correlated with the levels of AREG.

**Conclusion:**

The present findings suggest that AREG functions to stimulate synovial cells and that elevated levels of AREG may be involved in the pathogenesis of RA.

## Background

Rheumatoid arthritis (RA) is a chronic inflammatory disease that is mainly characterized by synovial hyperplasia and progressive destruction of the affected joints. Activated synoviocytes in the hypertrophic synovia induce angiogenesis, and play pivotal roles in the recruitment and differentiation of inflammatory cells. However, the driving force of the synovial hyperplasia remains obscure.

The granulomatous tissues of RA synovia, referred to as pannuses, resemble tumors. Cultured fibroblast-like synoviocytes (FLS) from these pannuses share some features with transformed cells, *i.e*. anchorage-independent growth [1,2] and downregulation of tumor suppressors [3-5]. Similar to transformed cells, tyrosine-phosphorylated proteins are augmented in RA-FLS, and several growth factors whose receptors possess tyrosine kinase activities have been reported to promote the tumor-like behavior of RA synovial membranes [6-9]. Since platelet-derived growth factor (PDGF) and fibroblast growth factor (FGF) stimulate DNA synthesis and proliferation of FLS cultured in medium containing low concentrations of serum [10] and histochemical studies have revealed upregulated expression levels of PDGF and FGF and their receptors in RA synovial tissues [11-13], these molecules are considered to be the major contributors to synovial hyperplasia [2,14].

On the other hand, the proto-oncogene c-erb-B, referred to as epidermal growth factor (EGF) receptor (EGFR), is a well-known tyrosine kinase growth factor receptor. Four members of the EGFR family have been identified to date, namely c-erb-B/EGFR and its related products ErbB2, ErbB3 and ErbB4. The family members form homodimers or heterodimers in various combinations, and exhibit different ligand specificities for the 13 members of the EGF family [15]. Although expression of ErbB2, but not the other ErbB-related receptors, has been reported to be augmented in RA synovial tissues [7,16,17], it remains unknown which members of the EGF family are expressed in the affected joints and involved in the pathology of RA.

In previous studies, we investigated the involvement of bone marrow in the pathology of RA. An increase in myeloid cells expressing abnormal surface antigens in bone marrow was associated with the severity of RA [18-23]. Pathogenic synovial fibroblasts may be derived from bone marrow CD34^+ ^cells in RA [24]. Recently, we identified RA-associated genes in bone marrow cells using a cDNA subtraction technique [25]. In that report, we demonstrated that two EGF-like growth factors, amphiregulin (AREG) and epiregulin (EREG), were upregulated in RA bone marrow.

In the present study, we examined the extents of involvement of EGF family members in RA pathology by investigating the expression of seven major EGF-like growth factors, namely EGF, AREG, EREG, transforming growth factor α(TGFα), heparin-binding EGF-like growth factor (HB-EGF), betacellulin (BTC) and neuregulin-1 (NRG1), in synovial tissues and mononuclear cells isolated from bone marrow and venous blood. The results revealed that AREG expression was augmented in all RA tissues and cells examined. Moreover, the AREG protein concentration in RA synovial fluid was significantly higher than that in osteoarthritis (OA) synovial fluid. Recombinant human AREG stimulated RA-FLS to proliferate and express several proinflammatory cytokines. These findings suggest that AREG may play a role in the pathogenesis of RA.

## Methods

### Patients and samples

Bone marrow fluid, venous blood and/or synovial tissues were intraoperatively obtained from 15 RA patients (all women; mean age ± SD: 59.3 ± 8.7 years) and 12 OA patients (all women; mean age ± SD: 64.5 ± 11.8 years) undergoing joint arthroplasty. None of the patients had taken any medication for at least 1 week before the operation. The RA and OA patients fulfilled the 1987 revised criteria of the American College of Rheumatology for the classification of RA [26] and the diagnostic criteria for OA [27], respectively. Bone marrow fluid and venous blood were mixed with heparin and separated by centrifugation at 1700 *g *for 15 min. After removal of the plasma, the blood cells and bone marrow cell fractions were adjusted to their original volumes with Hank's balanced salt solution (HBSS) and fractionated by density-gradient centrifugation at 3000 *g *for 30 min on Ficoll-Hypaque (GE Healthcare Bioscience, Tokyo, JPN). Mononuclear cells were collected from both the bone marrow and peripheral blood and used for the experiments described below. For further separation, the collected mononuclear cells were fractionated by magnetic beads coated with immobilized CD14, CD3 or CD19 antibodies (Miltenyi Biotec, Tokyo, JPN), since CD14, CD3 and CD19 are lineage-specific markers for monocytes, T lymphocytes and B lymphocytes, respectively. The cell populations fractionated by these antibodies were measured using flow cytometry, and confirmed to be > 95% pure. Synovial fluid was obtained from 24 RA patients and 10 OA patients and venous blood was obtained from 57 RA patients and 12 OA patients attending the outpatient clinic of our hospital. Synovial fluid was separated from cells and debris by centrifugation, and the clear supernatant was collected. Plasma was collected by centrifuging heparinized blood as described above. The synovial fluid and plasma samples were analyzed by ELISAs. All patients and healthy volunteers provided informed consent for participation in the study, which was approved by the Ethical Committee of the National Hospital Organization, Sagamihara National Hospital.

### Isolation of FLS and establishment of cell lines

Synovial membranes were minced aseptically and then digested enzymatically with 1 mg/ml collagenase (Wako, Osaka, JPN) in Dulbecco's modified Eagle's medium (DMEM; GIBCO, Grand Island, NY, USA) for 2 h at 37°C. Single cell suspensions were filtered through a nylon mesh, seeded in culture dishes containing DMEM supplemented with 100 units/ml penicillin, 0.1 mg/ml streptomycin (GIBCO) and 10% fetal bovine serum (FBS; Hyclone, Logan, UT, USA), and cultured at 37°C in humidified air containing 7.5% CO_2_. Since freshly isolated FLS contain many lymphocytes, monocytes and granulocytes, we used homogeneous fibroblastic cell populations after more than 4 passages. In proliferation assays, FLS cultures were stimulated by various concentrations of recombinant human AREG (R&D Systems, Tokyo, JPN) for 2 days. Prior to cell harvesting onto glass fiber disks, FLS were cultured with ^3^H-thymidine for 18 h. The radioactivities on the disks were measured using a liquid scintillation counter.

### RNA extraction and cDNA synthesis

Total cellular RNAs were extracted using the TRIZOL™ reagent (Invitrogen, Tokyo, JPN) according to the manufacturer's instructions. For RNA extraction from synovia, minced tissues were homogenized in TRIZOL using a Polytron homogenizer and the extracted RNAs were further purified using an RNeasy micro kit (QIAGEN, Tokyo, JPN). In cytokine induction assays, FLS cultures were stimulated by various concentrations of recombinant human AREG and/or genistein (SIGMA, Tokyo, JPN) for 3 h and subjected to RNA extraction using the RNeasy micro kit. First-strand cDNAs were synthesized from 2 μg of total RNAs by priming with oligo dT and Omniscript™ reverse transcriptase (QIAGEN) according to the manufacturer's instructions.

### Quantitative RT-PCR

Using real-time PCR, we estimated the mRNA expression levels of four EGFR family members and seven EGF family members. In subsequent investigations, the mRNA expression levels of five proinflammatory cytokines, namely interleukin (IL)-1β, IL-6, IL-8, tumor necrosis factor-α (TNF-α) and granulocyte-macrophage colony-stimulating factor (GM-CSF) were measured. The mRNA expression levels of vascular endothelium growth factor (VEGF), platelet-derived growth factor (PDGF), basic fibroblast growth factor (bFGF), a disintegrin and metalloproteinase (ADAM) 10 and ADAM17 were also measured. The primer sequences used were: 5'-GTGATTCCATCATGTATCCCAGGAG-3', 5'-AGATGCACTGTCCATGCAAACAA-3' (EREG); 5'-CTTCACTGTGTGGTGGCAGATG-3', 5'-ATGCAGTAATGCTTGTATTGCTTGG-3' (BTC); 5'-CAACCAGTGGCTGGTGAGGA-3', 5'-GAGCCCTTATCACTGGATACTGGAA-3' (EGF); 5'-GTGGTGCTGTCGCTCTTGATACTC-3', 5'-TCAAATCCATCAGCACTGTGGTC-3' (AREG); 5'-GGGCATGACTAATTCCCACTGA-3', 5'-GCCCAATCCTAGACGGCAAC-3' (HB-EGF); 5'-AGATAGACAGCAGCCAACCCTGA-3', 5'-CTAGGGCCATTCTGCCCATC-3' (TGFα); 5'-AGAATGTGCCCATGAAAGTCCAA-3', 5'-GCAGATGCCGGTTATGGTCAG-3' (NRG1); 5'-GGTGCGAATGACAGTAGCATTATGA-3', 5'-AAAGGTGGGCTCCTAACTAGCTGAA-3' (EGFR); 5'-CAGGCACCGCAGCTCATCTA-3', 5'-TCCCAGGTCACCATCAAATACATC-3' (ErbB2); 5'-CCCAGCATCTGAGCAAGGGTA-3', 5'-TTTAGGCGGGCATAATGGACA-3' (ErbB3); 5'-TGATAGGCCGTTGGTTGTCTGA-3', 5'-CCAGGTAGACATACCCAATCCAGTG-3' (ErbB4); 5'-CCCACTGAGGAGTCCAACAT-3', 5'-AAATGCTTTCTCCGCTCTGA-3' (VEGF); 5'-CTCTGATCATGCTAATGGCTGGA-3', 5'-GCTGCAGTTAGCGTCTCATGTGT-3' (ADAM10); 5'-GTGACATGAATGGCAAATGTGAG-3', 5'-AGACCCAACGATGTTGTCTGCTA-3' (ADAM17); 5'-CCCCTGCCCATTCGGAGGAAGAG-3', 5'-TTGGCCACCTTGACGCTGCGGTG-3' (PDGF); 5'-GTTGTGACAACCACAAGCAC-3', 5'-CTCTCACACTATCCACTGGT-3' (bFGF); 5'-ACACTGCGCCAACACAGAAATTA-3', 5'-TTTGCTTGAAGTTTCACTGGCATC-3' (IL-8); 5'-AAGCCAGAGCTGTGCAGATGAGTA-3', 5'-TGTCCTGCAGCCACTGGTTC-3' (IL-6); 5'-CCAGGGACAGGATATGGAGCA-3', 5'-TTCAACACGCAGGACAGGTACAG-3' (IL-1β); 5'-CATGATGGCCAGCCACTACAA-3', 5'-ACTGGCTCCCAGCAGTCAAAG-3' (GM-CSF); 5'-GACAAGCCTGTAGCCCATGTTGTA-3', 5'-CAGCCTTGGCCCTTGAAGA-3' (TNF-α). Real-time PCR was performed using a LightCycler 2.0 (Roche Diagnostics, Tokyo, JPN) and SYBR Premix Ex Taq (Takara, Kyoto, JPN) following the manufacturers' protocols. The amounts of PCR products were assessed by the fluorescence of SYBR Green intercalated in the DNA fragments, and melting curves were routinely recorded to verify the singularity of the products. The amplified products using each primer pair were cloned into the pGEM-T vector (Promega, Tokyo, JPN) and plasmids linearized by enzymatic digestion were used as quantification standards. A reference cDNA was used in every assay to control the precision among assays. The cDNA levels among the samples were normalized by the expression level of the internal control gene GAPDH (5'-GCACCGTCAAGGCTGAGAAC-3', 5'-ATGGTGGTGAAGACGCCAGT-3').

### ELISAs

The AREG protein concentrations in plasma samples from 57 RA patients, 12 OA patients and 9 healthy volunteers and synovial fluid samples from 24 RA patients and 8 OA patients were determined using an AREG Duo-set ELISA kit (R&D Systems). The protein concentrations of IL-1β, IL-6, IL-8, TNF-α, GM-CSF and VEGF in culture supernatants of RA-FLS stimulated with recombinant human AREG (R&D Systems) and those of VEGF and IL-8 in synovial fluid samples from 9 RA patients and 7 OA patients were determined using a Quantikine ELISA kit (R&D Systems).

### Statistical analysis

Statistical analysis was carried out using the StatView statistical analysis software (SAS, Cary, NC, USA). Differences between RA specimens and controls were determined to be significant when P < 0.05 by the Mann-Whitney U-test. The effects of AREG on RA-FLS were analysed by the Mann-Whitney U-test following the Kruskal-Wallis test. Correlation coefficients (ρ) were calculated by Spearman's rank correlation method and tested for statistical significance at the 0.05 (two-tailed) level.

## Results

### Expression profiles of EGF family members in bone marrow mononuclearcells (BMMCs)

**Figure 1:**
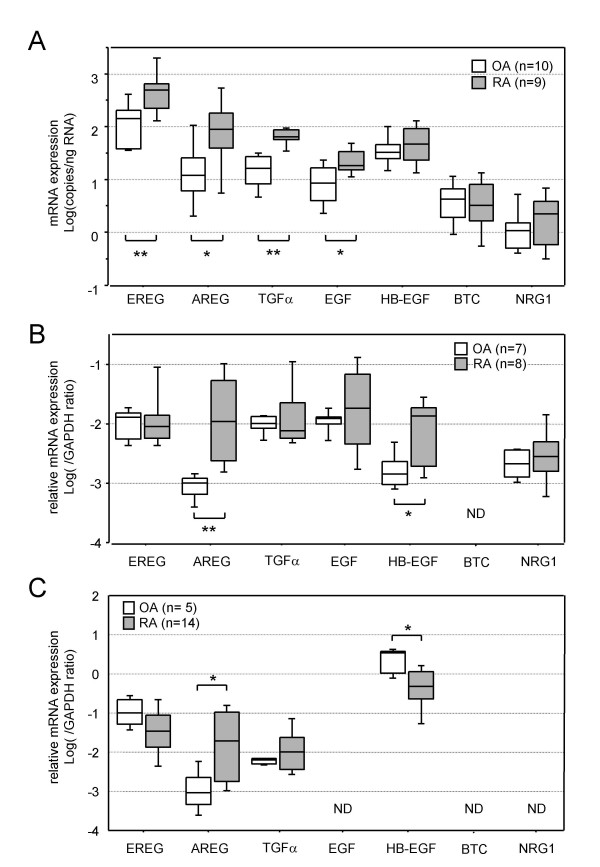
**mRNA expression levels of EGF-related growth factors in BMMCs (A), PBMCs (B) and synovial tissues (C)**. The results of real-time PCR are shown as box-plots. The log ratio of the mRNA quantities relative to the total RNA amount (A) or GAPDH mRNA (B, C) is plotted on the y-axis of each graph. The upper and lower error bars indicate the 90th and 10th percentiles, respectively. The upper and lower edges of each box indicate the 75th and 25th percentiles, respectively, and the line inside the box shows the median. Genes not detected are shown as ND. The differences between the mRNA levels in the RA and control samples were analyzed by the Mann-Whitney U-test, and significant differences are shown by asterisks (*P < 0.05; **P < 0.01). RA: samples from RA patients; OA: samples from OA patients.

First, we determined the mRNA expression levels of seven EGF family members in BMMCs obtained from 9 RA patients and 10 OA patients (Fig. 1A). EREG was the most abundantly expressed, and its mRNA level in RA-BMMCs was significantly higher than that in OA-BMMCs (P = 0.0060). The expression levels of AREG, TGFα and EGF were about 10-fold lower than that of EREG in OA-BMMCs, but significantly upregulated in RA-BMMCs (P = 0.0258, P = 0.000045 and P = 0.0140, respectively). Although the expression of HB-EGF was the next most abundant in RA-BMMCs, there was no significant difference between its expression levels in RA- and OA-BMMCs. The BTC and NRG1 mRNA expression levels were almost undetectable in both RA- and OA-BMMCs.

### Expression profiles of EGF family members in peripheral blood mononuclear cells (PBMCs)

Next, we determined the mRNA expression levels of the seven EGF family members in PBMCs obtained from 8 RA patients and 7 OA patients (Fig. 1B). EREG, TGFα and EGF were highly abundantly expressed in PBMCs, and their mRNA levels in RA- and OA-PBMCs did not differ. The expression levels of AREG, HB-EGF and NRG1 were about 10-fold lower than the levels of the highly abundant members in OA-PBMCs. Although AREG and HB-EGF were markedly upregulated in RA-PBMCs (P = 0.0017 and P = 0.0367, respectively), NRG1 was not upregulated in RA-PBMCs. BTC was not detected in either type of PBMCs.

### Expression profiles of EGF-like growth factors in synovial tissues

Next, we determined the mRNA expression levels of the seven EGF family members in synovial tissues obtained from 14 RA patients and 5 OA patients (Fig. 1C). Although HB-EGF was the most abundantly expressed and EREG was the next most abundantly expressed in synovial tissues from both RA and OA joints, their mRNA levels in RA synovia were somewhat lower than those in OA synovia. On the other hand, AREG expression, which was 1000-fold lower than HB-EGF expression in OA synovia, was markedly upregulated in RA synovial tissues (P = 0.0110). Expression of EGF, BTC or NRG1 was not detected in either OA or RA joints. Since only AREG expression was augmented in BMMCs, PBMCs and synovia of RA patients compared with the levels in control samples among the seven EGF-related growth factors examined, we narrowed the focus of the study to AREG.

### Determination of plasma and synovial fluid concentrations of AREG

**Figure 2:**
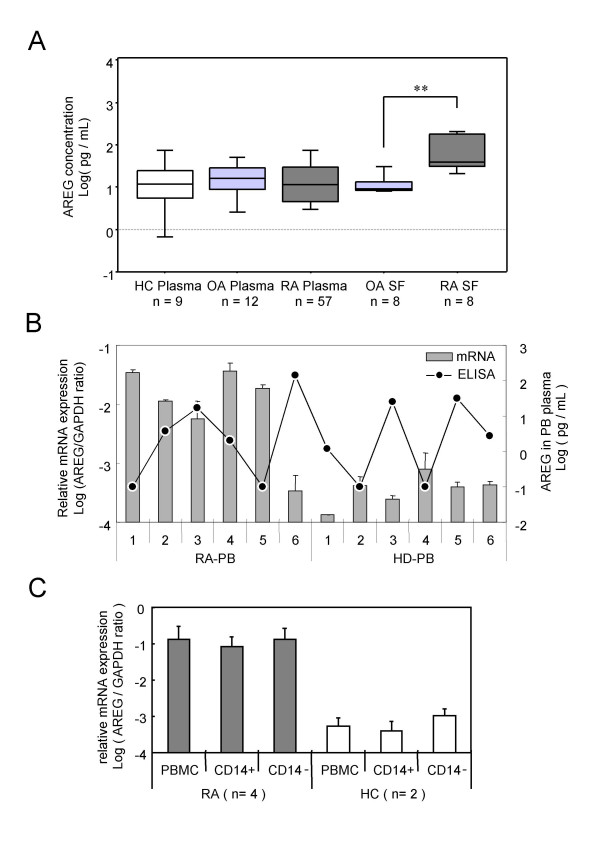
**Amphiregulin expression in peripheral blood and synovial fluid samples**. (A) The concentrations of AREG in plasma and synovial fluid samples are plotted as log-values on the y-axis of box-plots. Significant differences are shown by asterisks (**P < 0.01). RA: samples from RA patients; OA: samples from OA patients; HC: samples from healthy volunteers. (B) The AREG mRNA expression levels in PBMCs and AREG protein concentrations in plasma are shown. Venous blood samples from 6 RA patients and 6 healthy volunteers (HC) were separated into plasma and PBMCs. Total RNAs were extracted from PBMCs and subjected to cDNA synthesis. The AREG mRNA levels were measured by real-time PCR and normalized by the GAPDH mRNA levels. The relative AREG mRNA level relative to the GAPDH mRNA level is plotted as the log ratio on the primary y-axis (left), while the plasma concentration of AREG protein measured by ELISA is plotted as the log value on the secondary y-axis (right). The correlation coefficient (ρ) of the protein level in plasma to the mRNA level in PBMCs is -0.378 (P = 0.2104). (C) PBMCs from 4 RA patients and 2 HCs were separated by CD14 microbeads, and the AREG mRNA level in each fraction was measured by real-time PCR. The log ratio of the AREG mRNA level relative to the GAPDH mRNA level is plotted on the y-axis.

To confirm whether the protein concentration of AREG was augmented in RA patients, the AREG concentrations in plasma and synovial fluid samples were examined by ELISA. As shown in Fig. 2A, there were no significant differences among the AREG protein concentrations in the RA, OA and healthy control (HC) plasma samples, whereas the AREG concentration in RA synovial fluid samples was significantly higher than that in OA synovial fluid samples. Evaluation of BMMC, PBMC and plasma samples from 5 RA patients revealed that the AREG mRNA levels in RA-PBMCs were highly correlated with those in RA-BMMCs (data not shown), but not correlated with the plasma concentrations of this protein (Fig. 2B). Besides transformed cells, AREG-producing cells were previously reported to be activated monocytes [28] and activated T lymphocytes [29]. To clarify which lineage of blood cells expressed AREG in RA, PBMCs from RA patients were fractionated using magnetic beads coated with immobilized CD14, CD3 or CD19 antibodies. As shown in Fig. 2C, both CD14-positive and CD14-negative fractions of RA-PBMCs expressed equal amounts of AREG mRNA, and their levels were markedly higher than that in control PBMCs. The CD3 and CD19 separations yielded similar results (data not shown).

### Effects of AREG on the proliferation of RA-FLS

**Figure 3:**
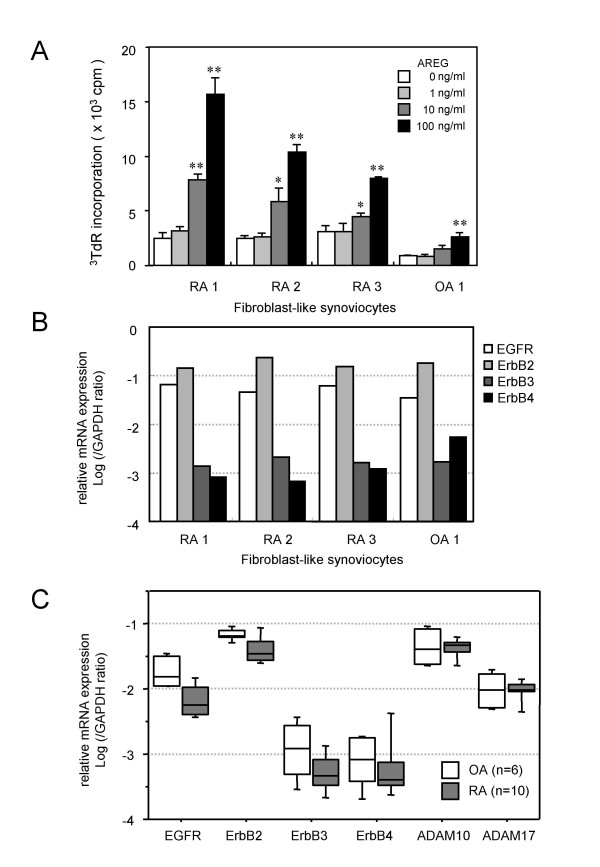
**Stimulatory activity of AREG on the proliferation of RA-FLS**. (A) Effect of AREG on the proliferation of RA-FLS. Three RA-FLS lines and one OA-FLS line were cultured with the indicated concentrations of AREG. After 24 h of stimulation, the cells were labeled with ^3^H-thymidine for 18 h and then harvested on glass filters with a cell harvester. The incorporated radioactivity was measured by liquid scintillation counting. The values are shown as the means ± SD of three independent experiments. Significant differences are shown by asterisks (*P < 0.05; **P < 0.01). (B) Expression profiles of EGFR family members in FLS. cDNA samples of the four FLS lines used in the proliferation assay were subjected to real-time PCR analysis. (C) Expression profiles of the receptors and sheddases of the EGF family in synovia. cDNAs of synovial tissues from 10 RA patients and 6 OA patients were subjected to real-time PCR analysis.

To investigate the biological activity of AREG in joints affected by RA, we assessed the effects of recombinant human AREG on RA-FLS. Since AREG is a member of the EGF-like growth factor family, its growth-promoting activity was measured first. As shown in Fig. 3A, recombinant human AREG enhanced *de novo *DNA synthesis by RA-FLS in a dose-dependent manner. Fig. 3B shows the expression levels of the four EGFR family members in the cell lines used in the proliferation assay. In all FLS cell lines, ErbB2 and EGFR were the predominantly expressed receptors and ErbB3 and ErbB4 were expressed at about 100-fold lower levels than the most abundant ErbB2 level. There were no differences among the three RA-FLS lines and the one OA-FLS line. Although the amounts of radioactivity incorporated into the RA-FLS lines were higher than that incorporated into the OA-FLS line, the issue of whether RA-FLS are more sensitive to AREG than OA-FLS requires further examination. EGF-like growth factors are expressed as transmembrane-type precursors, and ectodomain shedding by ADAMs is essential for their effects as well as the expression of their receptors [30,31]. Since ADAM10 and ADAM17 are known to be sheddases for EGF-like growth factors, we measured the expression levels of the four EGFR-like receptors and ADAM10 and ADAM17 in synovial tissues from 10 RA patients and 6 OA patients (Fig. 3C). Similar to the findings for FLS, EGFR and ErbB2 were the predominantly expressed receptors in synovial tissues and their expression levels were not augmented in RA samples. Although AREG is supposed to be mainly processed by ADAM17, ADAM17 was expressed at a lower level than ADAM10, and neither ADAM10 nor ADAM17 was upregulated in RA synovia.

### Effects of AREG on cytokine production by RA-FLS

**Figure 4:**
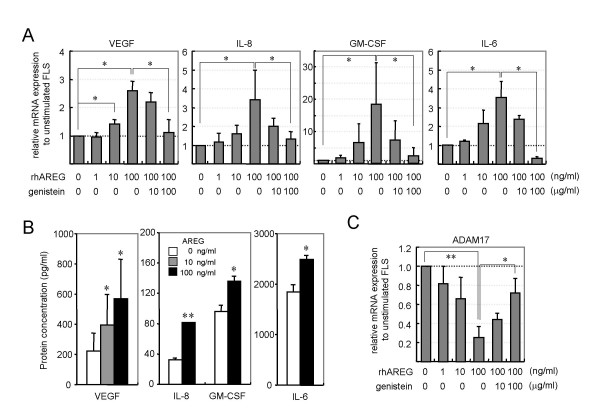
**Stimulatory activity of AREG on cytokine production by RA-FLS**. (A) Effects of AREG on cytokine expression in RA-FLS. Four RA-FLS lines were cultured with the indicated concentrations of recombinant human AREG and/or genistein. After 4 h of stimulation, total RNAs were extracted and the mRNA levels of PDGF, bFGF, VEGF, IL-1β, IL-6, IL-8, TNF-α and GM-CSF were measured by real-time PCR. The results for VEGF, IL-8, GM-CSF and IL-6 are shown. The results for the other molecules were omitted from the figure, since AREG had no effect on their expressions. (B) Effects of AREG on cytokine production by RA-FLS. Four RA-FLS lines were cultured with the indicated concentrations of AREG for 24 h. The GM-CSF, IL-6, IL-8 and VEGF concentrations in the supernatants were measured by ELISA, and are shown as means ± SD. (C) Effects of AREG on the expression of sheddases. The same cDNA samples used in panel A were subjected to real-time PCR analysis for ADAM10 and ADAM17. The results for ADAM10 were omitted from the figure, since they were similar to those for ADAM17. Each panel shows a representative result of three independent experiments. Significant differences from unstimulated cells are shown by asterisks (*P < 0.05; **P < 0.01).

Next, we tested the expression levels of five proinflammatory cytokines (IL-1β, TNF-α, IL-8, GM-CSF and IL-6) in RA-FLS stimulated by recombinant AREG. To clarify whether the recombinant AREG stimulated RA-FLS to proliferate via the induction of other growth factors, we also tested the expression levels of PDGF, bFGF and VEGF, which are involved in synovial hyperplasia. Recombinant AREG upregulated the mRNA expression levels of VEGF, IL-8, GM-CSF and IL-6 (Fig. 4A), but not those of PDGF, bFGF, IL-1β or TNF-α (data not shown). Recombinant AREG stimulated RA-FLS to express these cytokines in a dose-dependent manner, and the EGFR-tyrosine kinase inhibitor genistein suppressed the AREG-dependent expression in a dose-dependent manner. ELISA analysis revealed elevated levels of VEGF, IL-8, GM-CSF and IL-6 proteins in culture supernatants of AREG-stimulated RA-FLS (Fig. 4B), consistent with the results of the real-time PCR. We also tested the expression levels of ADAM10 and ADAM17 in AREG-stimulated RA-FLS. Contrary to the effect on the cytokine induction, AREG downregulated the expression of ADAM17 in a dose-dependent manner, and the AREG-dependent suppression was abolished by genistein (Fig. 4C). Analyses of ADAM10 expression produced similar results to those for ADAM17 (data not shown).

### Correlation between VEGF and AREG expression levels

**Figure 5:**
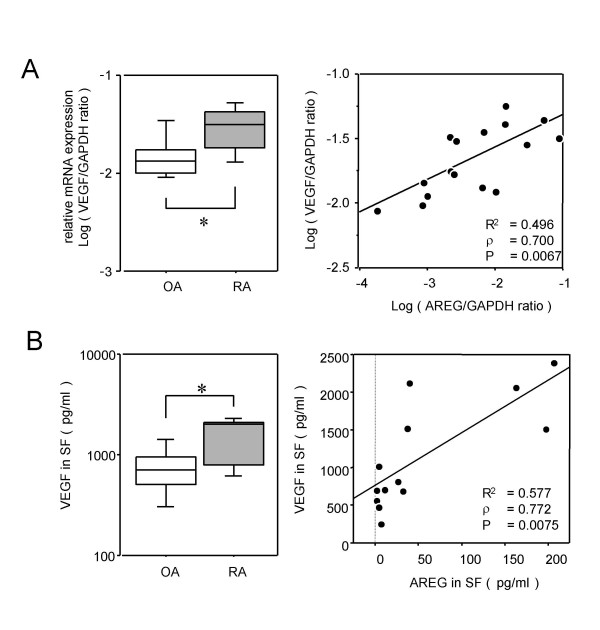
**Correlation between AREG and VEGF expression levels**. (A) The VEGF mRNA expression levels in synovia from 10 RA patients and 6 OA patients were measured by real-time PCR, and are shown by box-plots (left). Significant differences from unstimulated cells are shown by asterisks (*P < 0.05). The correlation between the mRNA levels of VEGF and AREG is shown by a distribution chart (right). The linear regression coefficient (R^2^) is 0.496 and the correlation coefficient (ρ) is 0.700 (P = 0.0067). (B) The concentrations of VEGF in synovial fluids from 7 RA patients and 6 OA patients were measured by ELISA, and are shown by box-plots (left). Significant differences from unstimulated cells are shown by asterisks (*P < 0.05). The correlation between the protein concentrations of VEGF and AREG is shown by a distribution chart (right), in which R^2 ^is 0.577 and ρ is 0.772 (P = 0.0075).

Since higher inductions of IL-6, IL-8 and GM-CSF than those induced by AREG have been observed and induction of VEGF has not yet been observed in our previous studies [32,33], we hypothesized that AREG would be closely related to VEGF in RA joints. To examine the relationship of AREG with this angiogenic factor in affected joints, VEGF expression was assessed in synovial tissues from 10 RA patients and 6 OA patients. Fig. 5A shows the mRNA levels of VEGF measured by real-time PCR. Ikeda et al. reported that the VEGF_165 _transcript may be augmented in RA synovial tissues, and that the products of this transcript may be associated with RA pathology [34]. The primers for the real-time PCR amplification of VEGF used in our study were also designed to detect VEGF_165_. VEGF expression in RA synovia was significantly higher than that in OA synovia (Fig. 5A, left panel), and highly correlated with AREG expression (Fig. 5A, right panel). Fig. 5B shows the VEGF protein concentrations in synovial fluid samples measured by ELISA. The ELISA system is able to detect all isoforms of VEGF-A, although it was designed for VEGF_165_. Consistent with the results of the mRNA expression analyses, the VEGF protein levels in RA synovial fluid samples were significantly higher than those in OA synovial fluid samples (Fig. 5B, left panel), and highly correlated with the AREG concentration (Fig. 5B, right panel).

## Discussion

Several previous studies have reported the involvement of c-erb-B family members, especially ErbB2, in the pathology of RA. Hallbech et al. showed immunohistochemically that the expression levels of ErbB2 and TGFα were augmented in RA synovia [17]. Satoh et al. demonstrated that ErbB2 was predominant in RA synovia and primary RA-FLS, but not in OA synovia or primary OA-FLS, and that a neutralizing antibody against ErbB2 suppressed the proliferation of primary RA-FLS, but not primary OA-FLS [16]. In the present study, we investigated which members of the ErbB family are predominantly expressed in RA-FLS and RA synovial tissues. Among the four ErbB family members, the mRNA level of ErbB2 was the highest, followed by that of EGFR, while the others were expressed at almost undetectable levels in FLS and synovial membranes. While these results are consistent with the previous report [16], there were no differences in the expression levels between RA and OA samples. This discrepancy may reflect differences in the synovial specimens, although it will be necessary to confirm this hypothesis by assessing the ErbB2 protein concentrations in RA and OA samples. The OA synovial samples used in the present study were obtained from synovia with villous formation, rather than from the joint capsule, and thus our OA samples may be more activated than those used in the previous study. In any case, ErbB2 and EGFR were confirmed to be predominantly expressed in RA-FLS and RA synovia.

On the other hand, there have been very few reports of the expression profiles of EGF-like growth factors in RA synovia. In the present study, we found that AREG expression, which was 1000-fold lower than the most abundantly expressed HB-EGF in OA synovia, was markedly upregulated in RA synovia. Since it was correlated with the expression of AREG (ρ = 0.532, P = 0.0241), the expression of TGFα may tend to be augmented in RA synovia, as reported previously [17].

Since we recently reported augmented expression of AREG in BMMCs and PBMCs from RA patients [25], we examined the expression levels of other EGF family members in the present study. In RA-BMMCs, EGF and TGFα were also upregulated, similar to AREG and EREG whose augmented expressions were reported in our previous study. Although there was no significant difference between the levels of HB-EGF in the RA and OA samples, its high correlations with AREG and EREG (ρ = 0.788, P = 0.0008 and ρ = 0.823, P = 0.0005, respectively) imply that HB-EGF may be upregulated in RA. Although EGF and TGFα were also upregulated in RA, their expression levels showed no correlations with those of other members. These results suggest that AREG, EREG and HB-EGF may be regulated by a common expression-controlling system.

In RA-PBMCs, AREG and HB-EGF were significantly upregulated, and their expression levels were correlated with each other (ρ = 0.600, P = 0.0305). Among the seven EGF family members examined, only AREG expression was augmented in all three tissues tested in the present study. To confirm which lineage of blood cells expresses AREG, PBMCs were further separated into monocyte-, T lymphocyte- and B lymphocyte-rich fractions. None of these fractions was enriched in AREG-expressing cells in healthy controls or RA patients. In our recent report, we speculated that bone marrow-derived abnormal monocytes expressing AREG may migrate via the blood circulation, and bring about disease in synovia and/or other tissues they infiltrated [25]. Although our present findings strongly support that hypothesis, the abnormal cells expressing AREG among RA-PBMCs were not restricted to monocytes. We conclude that they are mononuclear leukocytes and not of a particular lineage.

Herceptin, a specific inhibitor of ErbB2, was reported to suppress the proliferation of RA-FLS, but not OA-FLS, and augmented expression of ErbB2 was considered to be a major contributor to the autonomous proliferation of RA-FLS [16]. In the present study, EGFR and ErbB2 were found to be predominantly expressed in synovial tissues and cultured FLS, with no differences between their expression levels in RA and OA. Furthermore, the expression levels of ADAM10 and ADAM17, which are also important for the functions of EGF-like growth factors, showed no differences between RA and OA. On the other hand, the mRNA and protein levels of AREG were upregulated in RA synovial tissues. Furthermore, recombinant human AREG enhanced the proliferation of FLS in a dose-dependent manner. In our study, differences were detected for EGF-like growth factors between RA and OA synovia, but not for their receptors or sheddases. AREG induces tyrosine phosphorylation of EGFR and transduces a stronger signal when bound to EGFR/ErbB2 heterodimers [15]. It has been reported that synovitis with granulomatous hyperplasia occurs in AREG transgenic mice [35]. These findings suggest that overexpression of AREG may promote the proliferation of synoviocytes in affected joints of RA patients. We investigated whether recombinant AREG induced the expression of PDGF and bFGF, which are well-known growth factors for hyperplastic proliferation of RA-FLS. We found that AREG had no effects on the expression of these factors, suggesting that AREG did not stimulate RA-FLS to proliferate via these growth factors. However, AREG stimulated RA-FLS to express VEGF, an angiogenic factor involved in synovial hyperplasia.

A large number of reports have shown that RA-FLS produce proinflammatory cytokines when stimulated by various stimuli [36,37]. In addition, we previously reported that RA-FLS produce proinflammatory cytokines, such as IL-6, IL-8, GM-CSF, IL-1β and/or TNFα, when co-cultured with monocytes or lymphocytes [32,33]. Our analyses of cytokine production in the present study revealed that AREG enhanced the production of several proinflammatory cytokines (IL-6, IL-8 and GM-CSF) and VEGF in RA-FLS. Since they were suppressed by an EGFR tyrosine kinase inhibitor, the AREG-dependent induction of these cytokines seemed to occur via activation of EGFR/ErbB2. Interestingly, AREG downregulated the expression of ADAM10 and ADAM17 in a dose-dependent manner. These results suggest the presence of negative feedback regulation of ADAMs via AREG/EGFR signaling. To assess the involvement of AREG in the elevated expression of VEGF in affected joints of RA patients, the correlations between their mRNA levels and protein levels in synovial fluid samples were analyzed, and good correlations were found for both the mRNA levels (ρ = 0.700, P = 0.0067) and protein levels (ρ = 0.772, P = 0.0075). There have been very few reports about cytokine induction by AREG to date. The present results suggest that the increased level of AREG may be involved in the upregulation of VEGF in RA joints, and demonstrate, for the first time, that AREG stimulates RA-FLS to produce proinflammatory cytokines, including angiogenic factors. IL-8 has been reported to be an angiogenic factor as well as a chemoattractant factor [38,39]. Although several studies have recently shown that AREG plays important roles in hyperplasia or angiogenesis of skin diseases or tumors [40-43], the role of AREG in RA pathology remains unknown. Ma et al. speculated on the involvement of AREG in the angiogenesis of tumors [40]. Although it is known that EGF and TGFα are potent angiogenic mediators [44,45], the proangiogenic activity of AREG has not been directly determined to date. Its induction of angiogenic factors, such as IL-8 and VEGF, strongly suggests that AREG may be involved in the angiogenesis of synovial hyperplasia in affected joints of RA patients.

Myeloid cells expressing abnormal cell surface markers have been observed in RA bone marrow [18,19] and reported to be correlated with the disease severity [20-23]. Consistent with these reports, the present study revealed that several EGF-like growth factors were upregulated in RA bone marrow cells, suggesting that the onset and/or progression of chronic synovitis may be influenced by alterations to the bone marrow in RA patients.

## Conclusion

Among the seven EGF-like growth factors, AREG was upregulated in synovial tissues of RA patients. Recombinant human AREG stimulated RA-FLS to proliferate and produce several proinflammatory cytokines, including angiogenic factors. These results suggest that the elevated expression of AREG in synovial tissues may be involved in RA pathology containing synovial hyperplasia. AREG-expressing cells were observed in both the blood and bone marrow of RA patients as well as in RA synovial tissues. Abnormal leukocytes may lead to the upregulated expression of AREG in affected joints of RA patients.

## Competing interests

The authors declare that they have no competing interests.

## Authors' contributions

SY designed the study, carried out the experiments, analyzed the data and drafted the manuscript. SI, KK and YH carried out the RNA extractions and cDNA syntheses. RM and KT carried out the quantitative real-time PCR. NS performed the measurements of ^3^H-TdR incorporation into fibroblast-like synoviocytes. TM, TJ and TO participated in the study design and collection of clinical samples. NY, NF, TI and RS participated in the study design and coordination as well as editing of the manuscript. All authors have read and approved the final manuscript.
